# Long COVID in the context of social determinants of health

**DOI:** 10.3389/fpubh.2023.1098443

**Published:** 2023-03-28

**Authors:** Nada Lukkahatai, Tamar Rodney, Catherine Ling, Brittany Daniel, Hae-Ra Han

**Affiliations:** ^1^School of Nursing, Johns Hopkins University, Baltimore, MD, United States; ^2^Bloomberg School of Public Health, Johns Hopkins University, Baltimore, MD, United States

**Keywords:** long COVID, post-acute sequelae SARS-CoV-2 infection, social determinants of health, health disparities, health equality

## Abstract

The COVID-19 pandemic has been a challenge for the public health system and has highlighted health disparities. COVID-19 vaccines have effectively protected against infection and severe disease, but some patients continue to suffer from symptoms after their condition is resolved. These post-acute sequelae, or long COVID, continues to disproportionately affect some patients based on their social determinants of health (SDOH). This paper uses the World Health Organization's (WHO) SDOH conceptual framework to explore how SDOH influences long COVID outcomes.

## Introduction

The COVID-19 pandemic has created numerous challenges for the public health system and has highlighted health disparities. Since the first known case of COVID-19 coronavirus-induced atypical pneumonia was reported on November 16, 2019, in China (1), there have been over 626 million confirmed cases with over 6.6 million deaths worldwide as of October 20, 2022 (2). Black, Hispanic, and Asian people have been disproportionately affected by COVID-19 infection, hospitalization, and death (3). In April 2021, the US Food and Drug Administration approved multiple vaccines for COVID-19 prevention for the general population. Despite highly transmissible variants, COVID-19 vaccines are, in general, highly efficient in protecting against infection and severe disease (4–6), and the number of COVID-19 survivors has exponentially increased since the rollout of COVID vaccines.

Nevertheless, emerging evidence indicates that patients may continue to suffer from persistent post-infectious symptoms (e.g., fatigue, brain fog, chest or throat pain, or dyspnea) for more than 2 months (median 72 days) and might also have at least one unscheduled outpatient visit up to 6 months post-diagnosis (7, 8). The prevalence of these persistent symptoms is 50% among non-hospitalized patients and up to 87% among hospitalized patients (9–11). To this end, the Centers for Disease Control and Prevention (CDC) have described the COVID-19-related symptoms that last longer than 4 weeks as “Post-Acute Sequelae of COVID-19” or long COVID (12).

Common symptoms associated with long COVID include persistent fatigue, pain, difficulty sleeping, and brain fog (13–15). A systematic review of 47 studies published from January 1, 2020 to March 11, 2021 explored the frequency and variety of persistent symptoms following COVID-19 infection and revealed that the prevalent long COVID symptoms are fatigue (40%), shortness of breath or dyspnea (36%), sleep disorders or insomnia (29.4%), cognitive deficit (17.6%), and atypical chest pain (13.1%) (16); however, the follow-up period, measurement of symptoms, and patient care settings varied across the studies, with the majority of studies focused on individuals previously hospitalized with COVID-19.

Caring for persistent COVID-19 symptoms and complications after the acute period increases healthcare utilization and financial burden for the patients and the healthcare system (17). Vulnerable populations with underlying conditions and individuals with low socioeconomic status may not seek the necessary advanced care because of the increased cost of care and lack of health insurance. Additionally, those struggling with long COVID symptoms are more likely to miss work, leading to decreased job security and less access to health care through reduced availability of occupational health services (18), highlighting the importance of assessing and addressing the social needs of individuals with long COVID.

In 2022, the American Academy of Physical Medicine and Rehabilitation (AAPM&R) published several multidisciplinary, collaborative consensus guidance statements for the assessment and treatment of long COVID-related conditions (19–22). All guidance statements highlighted the importance of health equity to recommend the consideration of social determinants of health (SDOH) in assessing and managing COVID-related symptoms. CDC's interim guidance for evaluating and caring for post-COVID conditions also included recommendations to assess SDOH for patients with long COVID-19 (23). To increase our understanding of SDOH's role in long COVID, this paper explores current evidence on health outcomes in long COVID (e.g., time to symptoms resolution, health care utilization, and quality of life, etc.) in the context of the SDOH framework.

## Long COVID in social determinants of health context

The impact of unfavorable SDOH on acute COVID-19 outcomes is well documented (24–27). Multiple attempts have been made to identify vulnerable populations by examining the predictors of risk of infection and the severity of COVID-19. The following factors were identified as potential contributors to the disparity in exposure to the infection, morbidity, and mortality of COVID-19: race/ethnic differences, older age, living with multiple chronic conditions, genetic differences in Human Leukocyte Antigen (*HLA*) alleles, low socioeconomic status, and limited access to care (28–31).

The role of SDOH in the persistent symptoms of long COVID is less clear. Limited evidence has revealed contributing factors of long COVID. Some studies suggest certain conditions such as sex, age, and comorbidities may increase the risk for developing long COVID (32–36). In an attempt to understand the impact of SDOH on persistent symptoms, we used the World Health Organization (WHO)'s SDOH as a conceptual framework for this paper.

**Figure 1:**
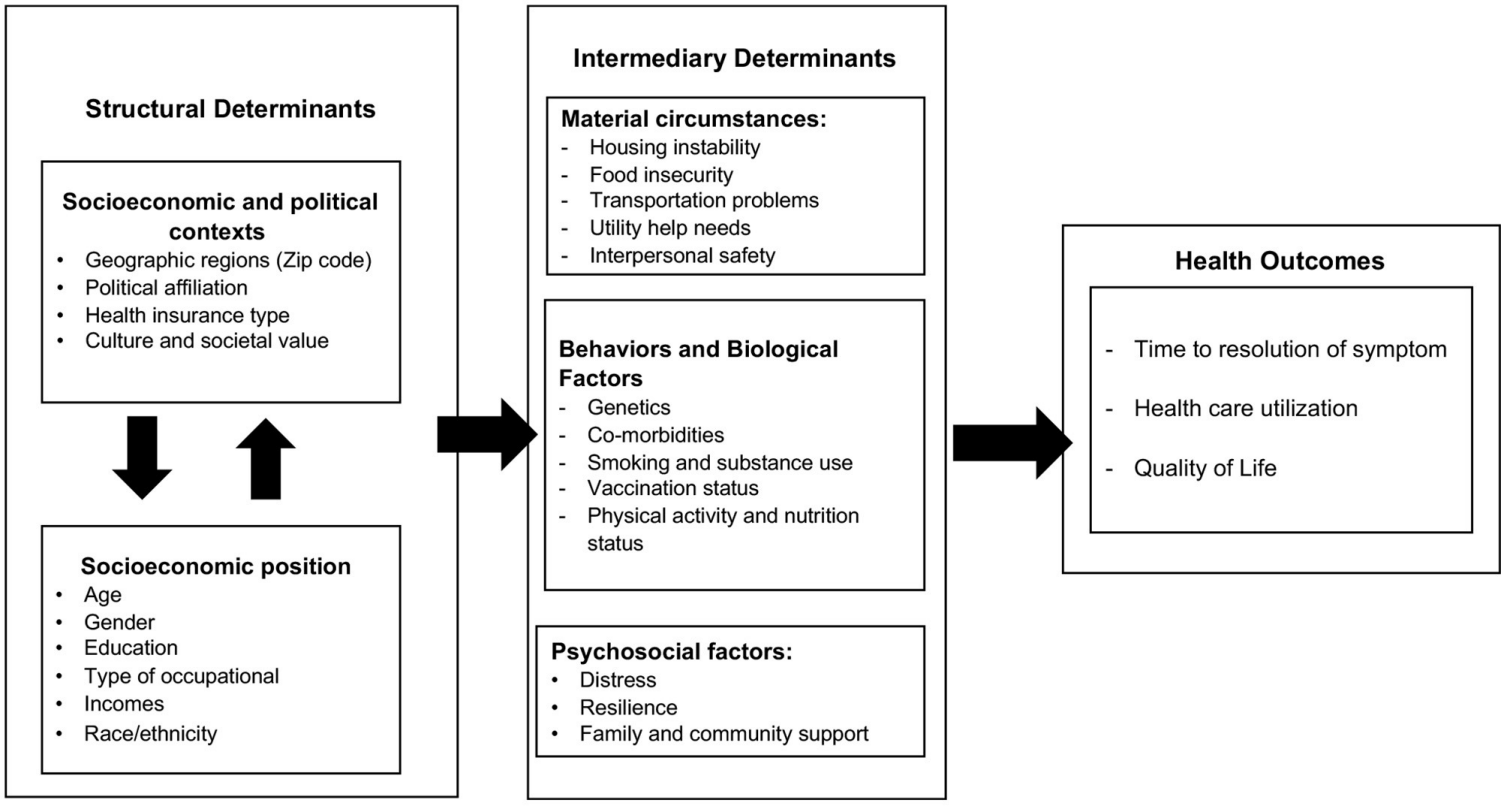
Long COVID health outcomes in the context of social determinants.

According to WHO, equity in health and wellbeing is impacted by two major elements: structural determinants and intermediary determinants (37). Adapting from the WHO's SDOH conceptual framework, long COVID-related health outcomes such as time to COVID-related symptoms resolution, healthcare utilization, and quality of life are impacted by structural and intermediary determinants (Figure 1).

S*tructural determinants* include (1) socioeconomic and political context, and (2) socioeconomic positions. The *socioeconomic and political contexts* are a broad set of structural, cultural, and functional aspects of a social system including governance, public and economic policies, culture, and societal values that affect health and affect an individual's health by interplay with the social structure (37). During the pandemic, evidence supported the role of geographic regions in COVID-19-related health outcomes. Cities and towns with high racially-based economic segregation, household crowding, and poverty had higher COVID-19-related mortality rates than predominantly white and wealthy suburban cities (38, 39). The beliefs, institutional trust, and political party affiliation have played key roles in most public health measures (e.g., social distancing, mandatory masks, and vaccination) to prevent the spread of COVID-19 (40–42). The results from US national survey data and a cohort study reported that individuals who self-identified as Republicans had the highest percent of “not wearing a mask,” infrequent physical distancing, and frequent visits to public indoor venues, restaurants/bars/clubs or attending or host parties with more than 10 people than Democrats and Independents (42, 43). Access to healthcare in low socioeconomic ethnic communities was the most significant predictor of high mortality rates related to COVID-19 (44).

The interplay between the socioeconomic and political contexts and social structure leads to social stratification or *socioeconomic positions* (37). In many societies, incomes, education and occupation, and race/ethnicity are proxies for the socioeconomic position (45). Factors such as sex and age play a role in long COVID as reported by studies that persistent COVID-19 symptoms are commonly reported by individuals identifying as female with an age range from 40 to 66 years (33, 34). For Black or African Americans, lower education rates and sociodemographic disadvantages (e.g., compact housing and lack of health care) are strong contributors to high COVID-19 cases and mortality rates (46, 47). Moreover, some occupations and industries are at higher risk for COVID-19 infection than others because of exposure to infections and proximity to others (48, 49). Therefore, the heightened risk of COVID-19 infection among these populations contributes to an increased risk for subsequent long COVID.

*Intermediary determinants* include three main categories: (1) material circumstances (e.g., housing instability, food insecurity, transportation problems, utility help needs, interpersonal safety); (2) health behaviors (e.g., smoking and substance use, vaccination status, physical activity, and nutrition) and biological factors (e.g., genetic and comorbidities); and (3) psychosocial circumstances (e.g., distress, resilience, and family/community support). Research has shown that the COVID-19 pandemic has had significant impacts on people of lower socioeconomic status and unfavorable material circumstances (50). During the pandemic, essential and low-income workers have had to continue to work in frontline roles, impacting their ability to comply with physical distancing and increasing their risk for exposure to COVID-19 (50). Additionally, food insecurity makes families unable to stockpile food supplies, thereby resulting in more frequent trips to supermarkets and increased risk of infection (50). Moreover, those experiencing housing instability are forced to rely on temporary living arrangements, often close to others, impacting their ability to physically distance and increasing their risk for COVID-19 infection (51). Finally, those with limited access to transportation must rely on public transit, which not only increases risk for exposure to COVID-19 but also limits access to health care due to transportation unreliability (52). Therefore, material circumstances influence one's risk for COVID-19 infection, in turn contributing to increased long COVID rates.

Evidence suggests that certain behaviors contribute to more severe acute COVID-19 infection and long COVID symptoms (53, 54). Smoking, for example, is known to be associated with poorer outcomes of acute COVID-19 (53). Using a new tool—“Post-COVID-19 Functional Status (PCFS) scale”—Hussein et al.'s (54) found statistically significant differences in functional restrictions related to smoking status, indicating more severe impairments among active smokers compared to former and non-smokers following COVID-19 infection. On the contrary, COVID-19 vaccination may protect against long COVID symptoms. In fact, decreasing or disappearing symptoms (e.g., fatigue, shortness of breath, insomnia, muscle pain, and gastrointestinal problems)—that is, symptom resolution—have been reported following at least one dose of a COVID-19 vaccine (55). Such symptom resolution is believed to be due to vaccine-induced T cells, stimulation of innate immune response, and the diversion of leukocytes, which cause long COVID (56). Pre-existing comorbid conditions (e.g., chronic inflammation, hypertension, cardiovascular disease, and diabetes) were the factors most associated with not returning to “usual health” after COVID (35, 36). Finally, possession of some genetic variations in Angiotensin-converting enzyme, apolipoprotein E, and Brain-Derived Neurotrophic Factors have also been identified as potential predictors for COVID-19 susceptibility and fatality (57–66).

Psychological factors also play a role in persistent symptoms. Not only is long COVID frequently associated with anxiety and depression, but these patients are also at risk for developing somatic symptom disorder due to excessive health-related thoughts as well as post-traumatic stress disorder (67). COVID-19 has increased the prevalence of mental health disorders and there is an expectation that this trend will also be reflected in individuals with long COVID symptoms (68, 69). These psychological outcomes are due in part to social isolation, concerns for family, and limited access to health services, which negatively impact mental health (70, 71). These factors, especially social isolation, and healthcare access may be worsened by material circumstances such as transportation restrictions and Wi-Fi inaccessibility. Additionally, the pandemic contributed to a rise in anti-Asian discrimination and pre-existing structural racism, such as wealth gaps and occupational segregation which disproportionately affect Black and Latinx people (72, 73). Although the prevalence of mental disorders and substance use does not differ among racial and ethnic minority groups, these groups are less likely to receive treatment (74). Furthermore, mental health may be worsened by hospitalization, as patients are exposed to additional risk factors, such as the side effects of treatments. Individuals who are exposed to increased stress in addition to having preexisting mental health vulnerabilities are at an increased risk for developing or worsening poor mental health outcomes.

Resilience has previously been explored as a buffer to negative health outcomes (75), and during the COVID-19 pandemic, it has reaffirmed that enhancing individual resilience can have a positive outcome for mental health conditions including depression (76–78). Although research has failed to find any statistical significance regarding resilience following acute COVID-19 infection, resilience may be a protective factor for the psychiatric outcomes of COVID-19 infection and long COVID (79). Moreover, resilience can be strengthened through the reduction of loneliness, which is a key contributor to distress and poor health (80). As we continue to gather evidence of the direct impact of long COVID-19 symptoms on mental health, primary care providers should be proactive in screening, referrals to local support services, and the provision of resources to target the increased negative impact related to individuals' SDOH (81–83).

*Health outcomes:* Individuals with long COVID may require specialized care, such as rehabilitation services, mental health counseling, pulmonary support, ongoing medication management, and monitoring of long-term health complications (84). Structural and intermediary determinants may play major roles in how individual seek help, use healthcare resources, and quality of life within the context of long COVID. For example, individuals with low socioeconomic status and low education who suffer from prolonged symptoms may not be aware of the long COVID symptoms or may have limited access to healthcare and social support, and face difficulty returning to work (85, 86). Some ethnic minority individuals with long COVID reported feeling stigmatized or dismissed by healthcare providers which contributed to delays in seeking care, inability to obtain disability benefits, and increased financial hardship (87). Dealing with prolonged health issues that impact living conditions, social support, employment status, and financial security may negatively influence an individual's quality of life.

## Discussion

The aim of this paper is to provide an initial assessment of available evidence that is consistent with the concepts in WHO's SDOH framework. We found evidence supporting the structural and intermediary determinants of long COVID outcomes. While the use of an established framework is a strength of this paper, the authors acknowledge that the overall interpretation and conclusion of this paper are potentially impacted by the following limitations. First, no inclusion and exclusion criteria were used for article selection, which could have led to selection bias. Second, the focus of this paper is on evidence in the United States. While other countries may face the challenges of addressing SDOH among individuals with long COVID, the response to these issues can vary significantly between countries. The recognition and response to SDOH can be influenced by a range of factors, including political will, cultural attitudes, historical context, and economic development. These limitations warrant extensive review of evidence and comparison of the perspectives on SDOH among countries.

## Conclusion

As we continue to see the new COVID-19 cases and mortality rate decline in 2022, the duration and recovery trajectories of COVID-related symptoms have yet to be clarified. Some patients with COVID will be disproportionally impacted by the long-term symptom burden. Ongoing support should remain a priority for these individuals, with ongoing reassessment and counseling as individuals discover and recover from long COVID-19 symptoms. Moreover, the recognition of emerging scientific evidence on long COVID-related symptoms and the impact of SDOH on long COVID could direct our efforts in promoting equity in the healthcare system. More research is needed to identify the time to symptoms resolution and contributing factors of long COVID-related outcomes, develop systematic and reliable measures for social determinants of health, and cultivate programs or interventions that can provide appropriate care and monitoring processes for patients with COVID who may be at risk of developing long COVID.

## Author contributions

NL wrote the editorial. TR, CL, BD, and H-RH participated in reviewing and editing the text. All authors contributed to the article and approved the submitted version.
